# The period effect in the prevalence of proliferative diabetic retinopathy, gross proteinuria, and peripheral neuropathy in type 1 diabetes: A longitudinal cohort study

**DOI:** 10.1371/journal.pone.0174979

**Published:** 2017-03-31

**Authors:** Christine A. Kiire, Kayla Horak, Kristine E. Lee, Barbara E. K. Klein, Ronald Klein

**Affiliations:** Department of Ophthalmology and Visual Sciences, University of Wisconsin School of Medicine and Public Health, Madison, Wisconsin, United States of America; University of Colorado Denver School of Medicine, UNITED STATES

## Abstract

**Aims:**

To investigate whether, for a specific duration of type 1 diabetes, there is a significant change in the prevalence of proliferative diabetic retinopathy, gross proteinuria and peripheral neuropathy in those more recently diagnosed with diabetes (a period effect), in the Wisconsin Epidemiologic Study of Diabetic Retinopathy. Where present, to determine how common risk factors for diabetic complications might be associated with it, and what might be driving it.

**Materials and methods:**

Longitudinal cohort study with seven examination phases between 1980 and 2014. Multivariate logistic regression models and ordinal parameterization were used to test for and evaluate any period effect.

**Results:**

There is a period effect in the prevalence of gross proteinuria and peripheral neuropathy (decreasing), as seen with proliferative diabetic retinopathy (p < 0.001). Adjusting for changing levels of common risk factors attenuates the period effect, particularly for proliferative diabetic retinopathy. For gross proteinuria and peripheral neuropathy, however there is a persistent period effect in spite of adjusting for the major risk factors.

**Conclusions:**

There are period effects in the prevalence of proliferative diabetic retinopathy, gross proteinuria and peripheral neuropathy that cannot be fully explained by changes in common risk factors for complications of type 1 diabetes in this cohort. The role of other potential confounders warrants further exploration.

## Introduction

Evidence suggests that for a specific duration of type 1 diabetes (T1D), the incidence, prevalence, and severity of diabetic retinopathy (DR) and diabetic nephropathy are lower in those with a more recent diagnosis [1–6]. This effect can be described as a “period effect”. It has generally been attributed to improvements in medical care [5], in particular, better glycemic and blood pressure (BP) control [2], although the details regarding the main contributors have yet to be expounded.

The Wisconsin Epidemiologic Study of Diabetic Retinopathy (WESDR) is one of very few long-term cohort studies of the complications of T1D. It is unique in providing over 30 years of follow-up data from a period beginning in 1980–1982. This makes it a valuable resource for assessing the impact of changes in common risk factors for microvascular complications of T1D on the prevalence of these complications.

We set out to investigate whether there is a period effect in the prevalence of gross proteinuria and/or peripheral neuropathy, as has previously been detected for DR in those with T1D in the WESDR. Where a period effect was present, we attempted to determine how it might be associated with the main risk factors for complications of T1D. To the best of our knowledge, this has not previously been evaluated in a comparable study.

## Materials and methods

This is a longitudinal cohort study that was approved by the institutional human subjects committee of the University of Wisconsin. It conforms to the tenets of the Declaration of Helsinki. Written informed consent was obtained from the study participants.

### Setting

The WESDR included 1210 persons with T1D living and receiving primary medical care in an 11 county area of southwestern Wisconsin from 1979–1980. Participants were invited to attend each of seven examinations starting from a period beginning in 1980–1982.

### Participants

For the primary analysis, adults with T1D and no history of dialysis or renal, pancreatic, or islet cell transplantation were eligible to participate in this study. Persons aged under 18 years were excluded because the risk factors for diabetic complications, and their management, may differ significantly between children and adults [7]. They could contribute once they reached 18 years of age. Persons requiring dialysis, and those who had undergone organ transplantation, were excluded from analysis beginning from the visit when they first reported having received these treatments. This was because of concern that their management at this stage might have affected important covariates, such as BP or glycosylated hemoglobin (HbA_1c_). They could contribute to the analyses prior to starting these treatments.

We performed secondary analyses that included those on dialysis and those who had undergone organ transplantation, in order to assess whether their exclusion from the primary analyses affected our findings significantly. Participants less than 18 years of age were still excluded from these secondary analyses until the visits where they were older.

### Variables

The complications selected for analysis were proliferative diabetic retinopathy (PDR), gross proteinuria, and peripheral neuropathy. PDR is a severe, vision-threatening form of DR. Other variables included in our analyses were duration of diabetes, sex, smoking status, body mass index (BMI), age at onset of diabetes, and the major risk factors for the complications being evaluated, that is, HbA_1c_ level and BP level [8–10]. We considered the role that serum total cholesterol might play. Duration of diabetes was calculated at each study visit and was stratified into categories ranging from <10 years to >30 years in 5-year increments. This 5-year spacing was selected because the examination phases were approximately 5 years apart.

Although, wherever possible, we obtained data on medication that participants were taking, as well as the methods that they were using to monitor blood glucose levels, the WESDR was not designed to assess the impact of these on the period effect. The changes in prescribing patterns and the technological advances in diabetes care that occurred over the timescale of the WESDR are tightly interwoven with the variables that were included in our analyses. The introduction of medication data would additionally lead to confounding by indication. It was therefore not possible to incorporate data on medication, such as the introduction of renin angiotensin system blockers and statins, and some of the improvements in diabetes management, such as the change from urine dipstick to finger-prick blood glucose monitoring, into our models in a way that would adequately reflect meaningful comparison groups. We believe that the majority of the effect of these changes is manifest through a lowering of the levels of the major risk factors which are being included in our analyses.

### Data sources

#### Proliferative diabetic retinopathy (PDR)

The prevalence of PDR at each study visit was determined by examination of 30-degree stereoscopic colour photographs of the retina for each eye, using Early Treatment Diabetic Retinopathy Study seven standard fields. The photographs were assessed by a grading protocol that has previously been described [11]. Participants with signs of scatter laser photocoagulation treatment for previous PDR were classified as having PDR in this study.

#### Gross proteinuria

Gross proteinuria was defined as a score of 1+ (≥ 30 mg/dL) on a random urine sample tested with Labstix (Ames, Elkhard, IN).

#### Peripheral neuropathy

A diagnosis of peripheral neuropathy was made on the basis of answers to the following specific questions:

“Since you were first told that you have diabetes, have you had any of the following problems? Do you have (or have you had):

- loss of sensation in your hands or feet?
- decreased ability to feel the hotness or coldness of things you touch?”

Participants answering “Yes” to either of these questions were considered to have peripheral neuropathy for the purpose of these analyses.

### Bias

The study cohort was fixed therefore survival bias may be present. We also know that persons with a longer duration of diabetes and more complications might be less likely to participate. This applies to all study visits, but the later examination phases, by definition, only include study subjects with a long duration of diabetes.

In the primary analyses, the exclusion of those on dialysis and those who had undergone organ transplantation, starting from the study visit when they first reported receiving these treatments, potentially introduces bias towards those with less severe nephropathy, less severe hypertension, and better control of their diabetes. For this reason, we performed secondary analyses with these participants included.

### Study size

The WESDR study size was determined by area-based recruitment eligibility criteria. The number of participants included in the analyses differed for each complication studied because it depended on the number of participants with a value assigned for each variable that was included in each statistical model.

### Statistical methods

SAS software (SAS Institute Inc., Cary, NC, Version 9.4) was used to perform all analyses. The period effect was assessed by testing the association of visit (treated as categorical) with the prevalence of PDR, gross proteinuria, and peripheral neuropathy, after adjustment for duration of diabetes in multivariate logistic regression models. The Type 3 test of association was used, and where significant, it indicates that the prevalence of the complication is significantly different for one visit compared to at least one other visit. Our threshold for significance, in each of our analyses, was *p* ≤ 0.05.

Ordinal parameterization was used to assess the change in the prevalence of each complication between consecutive visits. Beta estimates (log odds for the prevalence) were used to calculate the odds ratio (OR) and 95% confidence interval (CI) for the change in prevalence between visits. The estimated prevalence of the outcome of interest, after adjusting for duration of diabetes and any other factors present in the model, was shown with least squares means. Since data from each visit for eligible participants were included in the models, the generalized estimating equations approach was used to account for correlation between multiple assessments of the same person. An independent correlation structure was employed.

An assessment of whether the prevalence showed a trend from earlier study visits (lower visit number) to more recent visits (higher visit number) was performed by testing the association of visit (treated as continuous) with the prevalence of each complication, after adjusting for the duration of diabetes.

These analyses were first performed on a base model of duration of diabetes and visit, in order to determine whether there was a period effect. If present, each potential confounder was added to this base model. Any variables that were found to alter the period effect (model beta estimate changes by 10% or more), or to be significantly associated with the complication of interest, were included in the final model. We tested for an interaction between duration of diabetes and visit in each of the final models. Lack of participation, including loss to follow up, resulted in missing values. Missing values were automatically excluded from the analyses. The effect of age was not directly adjusted for because of collinearity with visit and duration of diabetes, which were already accounted for in the models.

## Results

### Participants

Of the 1210 persons originally identified as potential WESDR participants with T1D, some died before examination was possible, and others refused to participate or were lost to follow up. Table 1 shows the numbers in each of these categories. The number of those who contributed to the WESDR study across the seven examination phases represents a total of 5035 person-visits (996 at visit 1, 915 at visit 2, 816 at visit 3, 724 at visit 4, 593 at visit 5, 550 at visit 6, and 441 at visit 7).

**Table 1:** Dates of the WESDR examinations and numbers of participants with type 1 diabetes.

Visit number	1	2	3	4	5	6	7
Dates	1980–1982	1984–1986	1990–1992	1995–1996	2000–2002	2005–2007	2012–2014
**Number confirmed to have died before examination**	13	87	190	254	299	368	457
**Number eligible to participate in WESDR**	1197	1123	1020	956	911	842	753
**Number of those who were eligible to participate in WESDR but refused to participate or were lost to follow up**	201	208	204	232	318	292	312
**Number seen at each WESDR examination**	996	915	816	724	593	550	441
**Percentage of surviving subjects seen at each WESDR examination**	83.2%	81.5%	80.0%	75.7%	65.1%	65.3%	58.6%

WESDR—Wisconsin Epidemiologic Study of Diabetic Retinopathy

**Fig 1:**
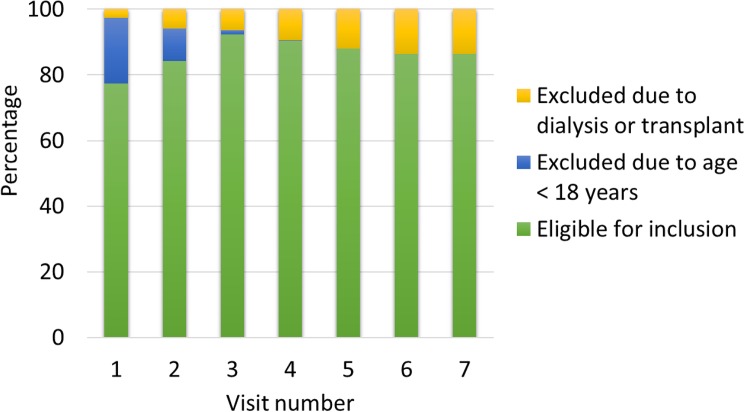
Proportion of potential participants who met inclusion and exclusion criteria, relative to the total number of people seen at each study visit.

Fig 1 shows the proportion of participants eligible for inclusion in the primary analyses, and the proportion that met our exclusion criteria, relative to the numbers that were seen at each visit. As might be expected, the main reason for exclusion from the earliest examination phases was age < 18 years, and by the most recent examination phases, where all participants were adults with a long duration of diabetes, the reasons for exclusion were a history of dialysis or organ transplantation. The number of participants who, depending on variable availability, had the potential to contribute to our analyses represents at total of 4327 person-visits (771 at visit 1, 770 at visit 2, 754 at visit 3, 654 at visit 4, 522 at visit 5, 475 at visit 6, and 381 at visit 7).

Table 2 shows the number of person-visits contributing to each model in the primary analyses, based on the availability of data on all of the variables included in each model. Data on the presence or absence of PDR was not available at visit 5 due to differences in photographic approach (the field definition was not comparable to the other examination phases). There was also a reduction in the percentage of participants with gross proteinuria, HbA_1c,_ BP, and BMI data at later study visits due to increasing numbers of remote examinations in which obtaining the laboratory measurements and measurements of physical characteristics, such as height, weight, and BP, was rarely feasible. The participants who opted for remote examinations tended to be those who were too unwell to travel to attend their study visit, or those who had moved out of the area, or were too busy to attend because of commitments such as work. Serum total cholesterol was first measured at the end of the visit 2 examination phase, in 45% of those who attended. The impact of changes in cholesterol levels could not, therefore, be explored to the same extent as the other risk factors.

**Table 2:** Number of person-visits contributing to each model in the primary analyses, based on availability of data on all of the variables included in each model.

Outcome	Base model		Final model	
	Number of person-visits included	Overall percentage of variable availability^a^	Number of person-visits included	Overall percentage of variable availability^a^
**Prevalence of proliferative diabetic retinopathy**	3619	83.6%	3120	72.1%
**Prevalence of gross proteinuria**	3570	82.5%	3298	76.2%
**Prevalence of peripheral neuropathy**	4313	99.7%	3507	81.0%

^a^Overall percentage of variable availability was calculated from the number of person-visits included in the model, divided by the total number of potential person-visits (4327) and multiplied by 100.

### Descriptive data

Selected characteristics of the person-visits eligible for the primary analyses, as well as the person-visits that were included in the final model of each outcome, are shown in Table 3. The characteristics of the person-visits with complete data for the final models (that is, those included in the analyses) were also compared with those of the person-visits with incomplete data for the final models (that is, those that could not contribute to the final models due to some variables lacking an assigned value), in order to highlight any significant differences between these groups.

**Table 3:** Selected characteristics of the all person-visits with the potential to contribute to the primary analyses and all person-visits with complete data for the final models.

Characteristics		All person-visits with the potential to contribute to the analyses		Person-visits with complete data which contributed to the final PDR model		Person-visits with complete data which contributed to the final gross proteinuria model		Person-visits with complete data which contributed to the final peripheral neuropathy model	
		n	mean (SD) or %	n	mean (SD) or %	n	mean (SD) or %	n	mean (SD) or %
**Age**	years	4327	40.4 (13.2)	3120	38.8 (13.2)**	3298	39.3 (13.2)**	3507	39.6 (13.1)**
**Duration of diabetes**	years	4327	25.5 (12.0)	3120	23.7 (12.0)**	3298	24.1 (11.8)**	3507	24.5 (11.8)**
**Age at diagnosis**	years	4316	14.8 (7.4)	3120	15.1 (7.4)**	3298	15.2 (7.5)**	3507	15.1 (7.4)**
**Sex**	% male	4327	48.6%	3120	49.7%*	3298	50.0%*	3507	49.8%*
**HbA1c**	%	3937	8.9 (1.8)	3120	9.1 (1.8)**	3298	9.1 (1.8)**	3507	9.0 (1.8)**
	mmol/mol		74		76		76		75
**MABP**	mmHg	3750	93 (12)	3119^a^	93 (12)	3298	93 (11)	3506^a^	93 (11)
**BMI**	kg/m^2^	3655	26.2 (4.8)	3120	26.1 (4.7)	3298	26.2 (4.7)	3507	26.3 (4.8)**
**Smoking history**	(% ever smoked)	4316	42.5%	3120	42.6%	3298	42.6%	3507	42.3%

WESDR—Wisconsin Epidemiologic Study of Diabetic Retinopathy, HbA1c - glycosylated hemoglobin, PDR—proliferative diabetic retinopathy, MABP—mean arterial blood pressure, BMI—body mass index

**Age-adjusted *p* ≤ 0.001 for the difference between those who had complete data and could contribute to the final model versus those who had incomplete data (due to missing values for variables) and could not contribute to the final model

*Age-adjusted *p* ≤ 0.05 for the difference between those who had complete data and could contribute to the final model versus those who had incomplete data (due to missing values for variables) and could not contribute to the final model

^a^Diastolic but not systolic blood pressure (which was included in the model) was missing for one participant

Strong similarities were seen in the levels of each characteristic that was assessed across the groups of those who contributed to the final models of PDR, gross proteinuria and peripheral neuropathy. These levels were close to those seen in the whole group, which includes those with incomplete data who, as a result of some variables not having an assigned value, could not contribute to the final models.

A more direct comparison between the person-visits with complete data and those without, shows statistically significant differences in age, and the age-adjusted levels of duration of diabetes, age at diagnosis (can be derived from the previous two variables), and HbA1c, for each outcome (p < 0.001 for each of these). Person-visits with incomplete data tended to involve participants who were older and had a longer duration of diabetes. They had, on average, been diagnosed with type 1 diabetes at a younger age. The proportion of males was higher in each group with complete data than it was in the groups with incomplete data.

### Outcome data

**Fig 2:**
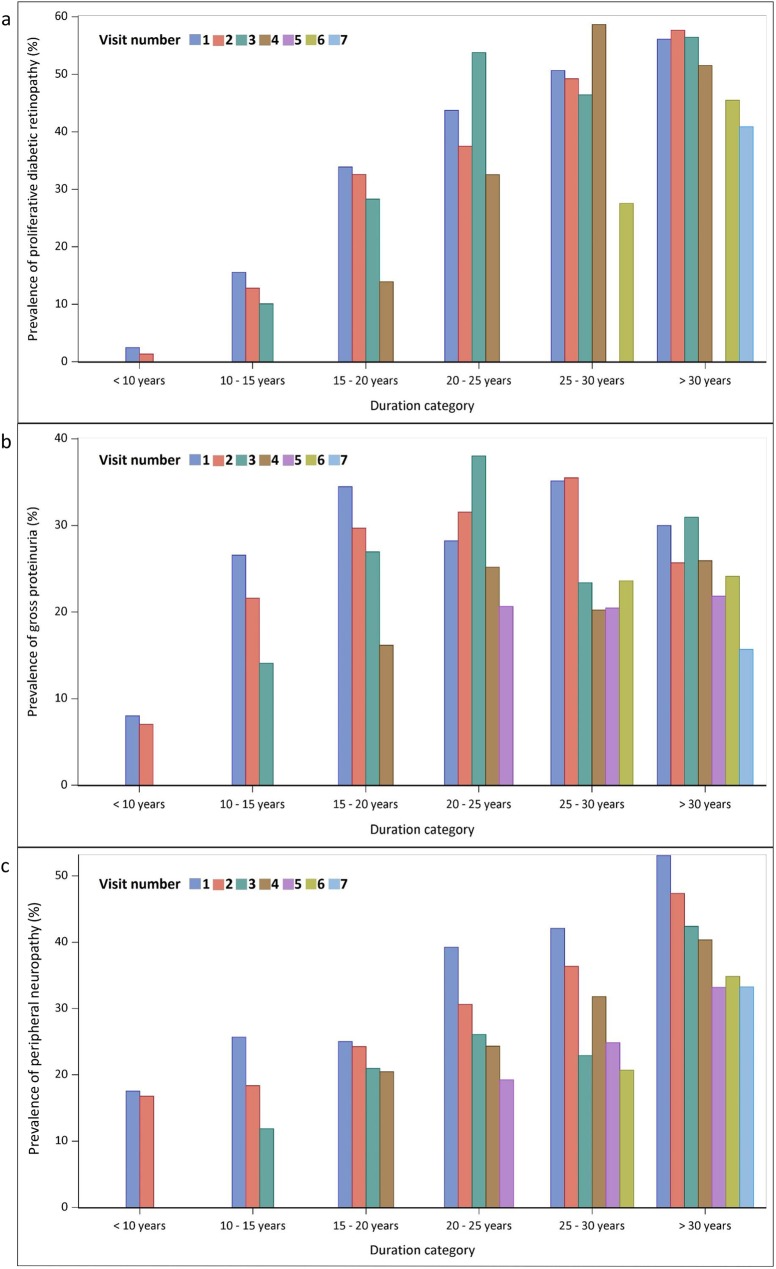
Prevalence of proliferative diabetic retinopathy, gross proteinuria, and peripheral neuropathy by diabetes duration and visit. (a) Prevalence of proliferative diabetic retinopathy. (b) Prevalence of gross proteinuria. (c) Prevalence of peripheral neuropathy.

The prevalence of PDR, gross proteinuria, and peripheral neuropathy, by duration, and across the study visits, from the primary analyses, is shown in Fig 2. There appears to be a period effect for each of these complications, as demonstrated by a downward trend in prevalence from the early study visits to the later study visits, within each category of diabetes duration.

### Main results–primary analyses

The logistic regression analyses confirm that there is a period effect in the prevalence of each of the selected complications when only the duration of diabetes and visit number are included in the model (PDR: *p* < 0.001, gross proteinuria: *p* < 0.001, peripheral neuropathy: *p* < 0.001). In these base models, the downward trend in prevalence is very similar for each of the complications (OR 0.86, 95% CI 0.81 to 0.92) (Table 4).

**Table 4:** Comparison between base and final models of proliferative diabetic retinopathy, gross proteinuria and peripheral neuropathy.

Outcome	Visit	Base model						Final model					
		Beta Estimate	Odds Ratio^a^	95% Confidence Interval		*p* value	Type 3 *p* value^b^	Beta Estimate	Odds Ratio^a^	95% Confidence Interval		*p* value	Type 3 *p* value^b^
				Lower	Upper					Lower	Upper		
**Proliferative diabetic retinopathy**	2 v 1	-0.12	0.89	0.75	1.06	0.20	<0.001	0.12	1.13	0.92	1.39	0.24	0.05
	3 v 2	0.05	1.05	0.88	1.24	0.60		-0.01	0.99	0.80	1.22	0.89	
	4 v 3	-0.35	0.70	0.61	0.81	<0.001		-0.23	0.79	0.67	0.94	0.01	
	6 v 4	-0.32	0.73	0.59	0.91	0.005		-0.08	0.93	0.70	1.23	0.60	
	7 v 6	-0.08	0.92	0.77	1.10	0.36		-0.14	0.87	0.70	1.08	0.20	
	Trend	-0.15	0.86	0.81	0.92	<0.001		-0.06	0.94	0.87	1.01	0.10	
**Gross proteinuria**	2 v 1	-0.15	0.86	0.71	1.06	0.16	<0.001	0.19	1.21	0.95	1.54	0.13	0.001
	3 v 2	-0.08	0.92	0.76	1.13	0.43		0.10	1.10	0.87	1.39	0.43	
	4 v 3	-0.37	0.69	0.56	0.86	0.001		-0.27	0.76	0.60	0.96	0.02	
	5 v 4	-0.08	0.92	0.67	1.28	0.63		-0.03	0.97	0.67	1.42	0.88	
	6 v 5	0.19	1.21	0.87	1.70	0.26		0.29	1.34	0.90	1.99	0.15	
	7 v 6	-0.54	0.58	0.41	0.84	0.004		-0.76	0.47	0.31	0.71	<0.001	
	Trend	-0.15	0.86	0.81	0.92	<0.001		-0.03	0.97	0.89	1.05	0.44	
**Peripheral neuropathy**	2 v 1	-0.23	0.80	0.66	0.96	0.02	<0.001	-0.13	0.88	0.70	1.10	0.26	0.009
	3 v 2	-0.31	0.73	0.60	0.89	0.002		-0.34	0.71	0.56	0.90	0.005	
	4 v 3	0.03	1.03	0.86	1.24	0.73		0.19	1.21	0.97	1.51	0.10	
	5 v 4	-0.31	0.73	0.59	0.91	0.005		-0.18	0.83	0.63	1.10	0.20	
	6 v 5	0.001	1.00	0.81	1.24	1.00		0.17	1.18	0.89	1.58	0.25	
	7 v 6	-0.03	0.97	0.77	1.21	0.76		-0.16	0.85	0.64	1.13	0.26	
	Trend	-0.15	0.86	0.81	0.91	<0.001		-0.07	0.93	0.87	1.00	0.05	

^a^The odds ratios have been calculated by exponentiating the beta estimates.

^**b**^The Type 3 *p* value indicates the probability that there is a statistically significant difference between at least two of the visits.

#### Proliferative diabetic retinopathy (PDR)

**Fig 3:**
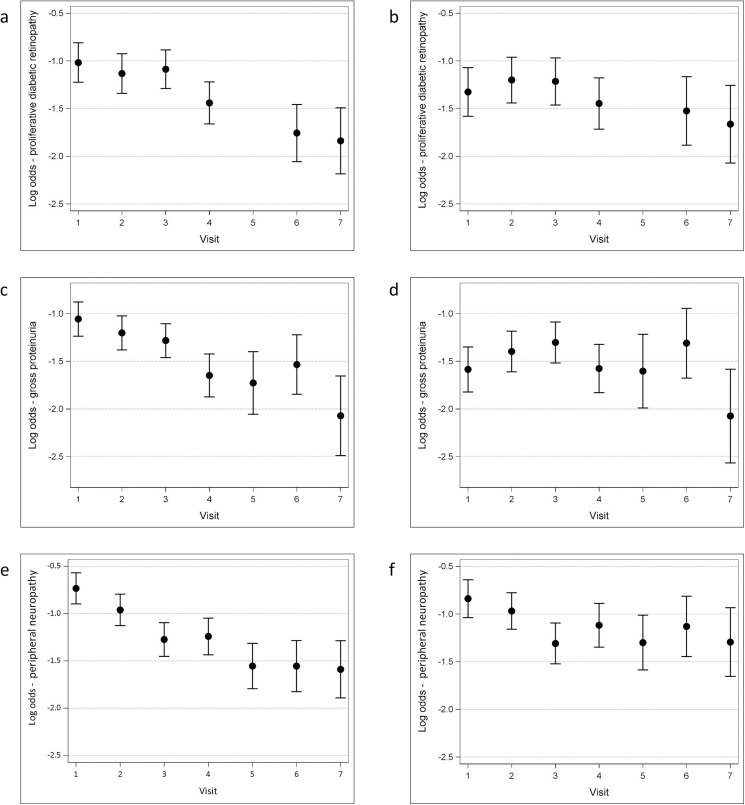
Adjusted estimated prevalence of each complication at each study visit. **(**a) Proliferative diabetic retinopathy–adjusted for duration of diabetes and visit. (b) Proliferative diabetic retinopathy–adjusted for duration of diabetes, visit, glycosylated hemoglobin, and systolic blood pressure. (c) Gross proteinuria—adjusted for duration of diabetes and visit. (d) Gross proteinuria—adjusted for duration of diabetes, visit, glycosylated hemoglobin, mean arterial blood pressure, and sex. (e) Peripheral neuropathy—adjusted for duration of diabetes and visit. (f) Peripheral neuropathy—adjusted for duration of diabetes, visit, glycosylated hemoglobin, sex, and smoking status. Log odds = log [p/(1-p)], i.e. log odds of -0.5 = prevalence of 38%, log odds of -1.0 = prevalence of 27%, log odds of -1.5 = prevalence of 18%, and log odds of -2.0 = prevalence of 12%.

In the base model of diabetes duration and visit number, the most significant reduction in the prevalence of PDR occurs between visits 3 and 4 (Fig 3A). Table 4 shows that there is also a significant reduction between visits 4 and 6.

The addition of HbA_1c_ to the base model eliminates most, but not all, of the period effect (*p* = 0.01), with the difference between visits 3 and 4 remaining significant. Systolic BP and BMI are associated with PDR but have less impact on the period effect. Diastolic BP and mean arterial BP have similar effects to systolic BP, but they are smaller in size. Sex, smoking status, and age at onset of diabetes are not significantly associated with PDR in this cohort, but smoking status and age at onset of diabetes confound the period effect.

The final model, therefore, comprises duration of diabetes, visit number, HbA_1c_, systolic BP, BMI, smoking status, and age at onset of diabetes (Fig 3B). This combination of variables can explain the period effect (*p* = 0.05), and the downward trend in the prevalence of PDR that was evident in the base model is no longer significant in this model (OR 0.94, 95% CI 0.87 to 1.01). We tested for an interaction between duration of diabetes and visit in this model and found that no interaction was present.

#### Gross proteinuria

Fig 3C shows the downward trend in the estimated prevalence of gross proteinuria across the study visits, when adjusting for diabetes duration. As seen with the prevalence of PDR, the most significant difference is between visits 3 and 4, but there is an additional significant difference between the prevalence of gross proteinuria at visits 6 and 7 (Table 4).

The period effect is reduced when HbA_1c_ is added to the model (*p* = 0.002). Although also significantly associated with gross proteinuria, the addition of BP variables to the visit and duration base model accentuates the period effect (*p* < 0.001, irrespective of which BP measure is considered). Mean arterial BP improves the fit of the model more than the other BP variables. It causes the reduction in prevalence from visit 6 to visit 7 to become more marked, but it attenuates the difference between visits 3 and 4. In this cohort, sex is significantly associated with gross proteinuria (risk higher in males), as is age at onset of diabetes. Smoking status and BMI are not significantly associated with gross proteinuria but are confounders of the period effect.

The final model for the prevalence of gross proteinuria therefore comprises duration of diabetes, visit, HbA_1c_, mean arterial BP, sex, age at onset of diabetes, smoking status, and BMI. It is unable to fully account for the period effect (*p* = 0.001), which is still mainly driven by the difference between visits 6 and 7, although the difference between visits 3 and 4 also remains significant (Fig 3D). As seen with the prevalence of PDR, there is no longer a significant downward trend in the prevalence of gross proteinuria in this model (Table 4). No interaction between duration of diabetes and visit was found.

#### Peripheral neuropathy

Modeling the period effect in the prevalence of peripheral neuropathy with only duration of diabetes and visit reveals a step-wise reduction in estimated prevalence across the study visits (Fig 3E). The most significant change in the prevalence of peripheral neuropathy occurs between visits 2 and 3, but other statistically significant differences are found between visits 1 and 2 and between visits 4 and 5 (Table 4).

The addition of HbA_1c_ to the base model causes a reduction in the period effect (*p* = 0.005). Male sex, smoking status and age at onset of diabetes are also significantly associated with peripheral neuropathy. BP and BMI are not significantly associated with peripheral neuropathy in this cohort but they confound the period effect.

The final model for peripheral neuropathy therefore includes duration of diabetes, visit, HbA_1c_, sex, smoking status, age at onset of diabetes, BP (systolic selected), and BMI. These variables cannot completely explain the period effect in the prevalence of peripheral neuropathy, but their inclusion in the model slightly reduces it (*p* = 0.009). The fall in the prevalence of peripheral neuropathy between visits 2 and 3 is the only significant difference that remains between the study visits with this model (Fig 3F). There is no longer a significant downward trend in the prevalence of peripheral neuropathy with this model (OR 0.93, 95% CI 0.87 to 1.00) and no interaction between duration of diabetes and visit was found.

### Other analyses

When we repeated the analyses with those on dialysis and those who had undergone organ transplantation (from the visits where they first reported receiving these treatments) included, the period effect was still present in the base models for all three complications (*p* < 0.001), with a similar pattern to that seen in the primary analyses. The updated final models comprised the same variables as already described, with the exception of PDR, which also included sex. In the final model for PDR there was an interaction between duration category and visit. This was not seen with the other models. The period effect could not be completely explained by the variables included for any of the complications studied (PDR: *p* = 0.02, gross proteinuria: *p* = 0.001, peripheral neuropathy: *p* = 0.01). As shown in Table 5, in the case of the prevalence of PDR, the period effect appeared to be driven by the difference in prevalence between visits 3 and 4. The period effects in the prevalence of gross proteinuria and peripheral neuropathy were driven by differences between the same visits as in the primary analyses. There was no longer a significant downward trend in the prevalence of PDR or gross proteinuria with the final models, but there was a persistent, although reduced, downward trend in the prevalence of peripheral neuropathy with the final model (*p* = 0.03).

**Table 5:** Comparison between base and final models of proliferative diabetic retinopathy, gross proteinuria and peripheral neuropathy when participants on dialysis or with kidney, pancreas or islet cell transplants are included.

Outcome	Visit	Base model						Final model					
		Beta Estimate	Odds Ratio^a^	95% Confidence Interval		*p* value	Type 3 *p* value^b^	Beta Estimate	Odds Ratio^a^	95% Confidence Interval		*p* value	Type 3 *p* value^b^
				Lower	Upper					Lower	Upper		
**Proliferative diabetic retinopathy**	2 v 1	-0.02	0.98	0.84	1.14	0.81	<0.001	0.14	1.15	0.95	1.39	0.14	0.02
	3 v 2	-0.05	0.95	0.81	1.11	0.52		-0.04	0.96	0.79	1.17	0.68	
	4 v 3	-0.30	0.74	0.66	0.84	<0.001		-0.24	0.79	0.67	0.92	0.002	
	6 v 4	-0.28	0.76	0.62	0.93	0.01		-0.09	0.92	0.71	1.19	0.52	
	7 v 6	-0.07	0.93	0.80	1.08	0.36		-0.11	0.89	0.73	1.08	0.25	
	Trend	-0.14	0.87	0.82	0.93	<0.001		-0.07	0.93	0.87	1.00	0.07	
**Gross proteinuria**	2 v 1	-0.14	0.87	0.71	1.05	0.15	<0.001	0.16	1.17	0.93	1.47	0.19	0.001
	3 v 2	-0.12	0.89	0.73	1.08	0.23		0.06	1.06	0.84	1.34	0.61	
	4 v 3	-0.34	0.71	0.59	0.87	<0.001		-0.28	0.75	0.60	0.95	0.02	
	5 v 4	-0.01	0.99	0.72	1.34	0.93		0.07	1.07	0.75	1.53	0.70	
	6 v 5	0.17	1.18	0.87	1.61	0.29		0.31	1.36	0.94	1.97	0.10	
	7 v 6	-0.49	0.61	0.44	0.84	0.003		-0.71	0.49	0.34	0.71	<0.001	
	Trend	-0.14	0.87	0.82	0.93	<0.001		-0.03	0.98	0.90	1.05	0.51	
**Peripheral neuropathy**	2 v 1	-0.17	0.84	0.71	1.01	0.06	<0.001	-0.10	0.90	0.73	1.11	0.34	0.01
	3 v 2	-0.35	0.70	0.59	0.84	<0.001		-0.31	0.74	0.59	0.91	0.005	
	4 v 3	0.03	1.03	0.87	1.21	0.73		0.13	1.14	0.93	1.40	0.21	
	5 v 4	-0.33	0.72	0.59	0.87	<0.001		-0.22	0.80	0.62	1.03	0.08	
	6 v 5	0.02	1.02	0.85	1.24	0.82		0.17	1.19	0.92	1.54	0.19	
	7 v 6	-0.02	0.98	0.81	1.20	0.87		-0.12	0.89	0.69	1.15	0.37	
	Trend	-0.15	0.86	0.82	0.91	<0.001		-0.07	0.93	0.87	0.99	0.03	

^a^The odds ratios have been calculated by exponentiating the beta estimates.

^**b**^The Type 3 *p* value indicates the probability that there is a statistically significant difference between at least two of the visits.

## Discussion

### Key results

These data suggest that there is a statistically significant period effect in the prevalence of each of the microvascular complications of T1D that were assessed in this cohort. The prevalence of each complication is generally lower in those with a more recent diagnosis when adjusting for duration of diabetes. In all cases apart from the prevalence of PDR when those who were on dialysis or had undergone organ transplantation were excluded, it was not possible to completely explain the period effect in terms of changes in the common risk factors for complications of T1D. The persistence of the period effect may be due to other contributing factors that are unaccounted for. When those on dialysis and those who have received organ transplants are included in the analyses, the increase in power allows us to detect more subtle differences in prevalence between the study visits. There may also be some impact of the additional treatments that those on dialysis, or who had received organ transplants, were receiving on the key variables included in the models.

### Strengths and limitations

This study spans more than three decades in which significant changes occurred in the management of diabetes. A period effect in the prevalence of PDR in T1D has previously been identified in the WESDR population [5]. The current analysis provides more detail on this period effect, including what might be driving it, over a longer period of time than was previously reported. In addition, this analysis provides novel data on period effects in the prevalence of gross proteinuria and peripheral neuropathy in the same cohort, over the same time period.

For practical reasons, we could not directly incorporate data on changes in diabetes management, in terms of medication prescribed and advances in methods for monitoring blood glucose, into our models. We do, however, believe that these changes have shown most of their impact through changes in the risk factors for complications of diabetes, providing us with a unique opportunity to examine how the prevalence of specific complications of T1D has been affected as a result. It remains possible that the medication that study subjects were taking, and other management changes that may have led to a reduction in the variability of blood glucose levels, might in some cases have had an impact beyond a simple lowering of the level of the risk factor they were designed to reduce. It was not possible for us to capture this type of impact with our analyses.

An important limitation in this study is that the population studied was almost entirely non-Hispanic white (99%), therefore it does not tell us about period effects in other ethnic groups. The lack of cholesterol data from the first two study visits meant that we could not fully explore the impact of this variable. Similarly, the absence of PDR data at visit 5 will have affected our ability to explore this fully. Wherever possible, we have commented on the trends visible in spite of the missing data. The participant characteristics shown in Table 3 suggest that the person-visits contributing to the final models for each complication studied (in which, by definition, there were no missing data) were very similar to those of the whole group of person-visits with the potential to contribute to the analyses.

There is a lack of consensus as to the most appropriate definition of diabetic nephropathy [8]. We chose to analyse gross proteinuria. Although it is commonly associated with diabetic nephropathy, we are aware that gross proteinuria is not specific to this condition. We were, however, unable to exclude participants with non-diabetic causes of gross proteinuria. We also acknowledge that our peripheral neuropathy variable does not distinguish between diabetic and non-diabetic causes of peripheral neuropathy, and that a significant proportion of cases of diabetic peripheral neuropathy may be asymptomatic [12], and therefore not detected with the questions that we asked the participants. This means that these variables, as defined in this study, might not fully reflect the precise prevalence of diabetic nephropathy or diabetic neuropathy. As gross proteinuria and peripheral neuropathy are key components of these diagnoses, however, we believe that it is still of value to present the analyses of these variables in spite of the limitations noted.

### Interpretation

In this cohort, a period effect is not just seen in the prevalence of PDR, but also in the prevalence of gross proteinuria and peripheral neuropathy.

In the case of PDR and gross proteinuria, the period effect appears to mainly be driven by a reduction in prevalence between visit 3 (1990–1992) and visit 4 (1995–1996). Several factors might have led to an improvement in HbA_1c_ and BP levels at this time. The National Eye Health Education Program was started in 1991 [13], and the results of the Diabetes Control and Complications Trial, emphasizing the importance of tight glycemic control, were published in 1993 [14]. Guidelines regarding the importance of tight BP control in T1D, particularly in relation to reducing the risk of diabetic nephropathy and reducing cardiovascular mortality, were issued in the 1980s [15]. The guidelines followed numerous studies which had highlighted the way in which hypertension increases the risk of diabetic complications, some of which were published around the time of first WESDR examination [16–18].

The persistent, significant reduction in the estimated prevalence of gross proteinuria between visit 6 (2005–2007) and visit 7 (2012–2014), in spite of adjusting for duration, HbA_1c_, BP, and sex, age at onset of diabetes, smoking status, and BMI, could, in part, be due to selective survival of those with the best renal function. The mean duration of diabetes was 41.8 years at visit 7, and 457 out of the originally identified population of 1210 potential participants were deceased by that time.

The period effect in the prevalence of peripheral neuropathy, as defined in this study, seems to be related to the reduction in prevalence between visit 2 (1984–1986) and visit 3 (1990–1992). There may be as yet undetermined, additional confounders influencing the prevalence of peripheral neuropathy over this time period.

### Generalizability

The results of this study are applicable to the cohort studied over this specific time period. They might, however, be more widely generalizable to other populations with similar demographics over the same period of time. The timing of changes in the risk factors for complications of T1D will vary according to rates of uptake of clinical guidance, health insurance coverage, and accessibility of diabetic care, including reliability of access to medication and access to screening for complications. The impact of these interventions over time might, however, be similar in other contexts. Future work in this area could include testing the repeatability of these findings in other populations over a similar time period, ideally with greater potential for assessing the impact of changes in serum cholesterol levels. Other methods of defining diabetic nephropathy could be considered, and a more objective measure of peripheral neuropathy (for example, the Michigan Neuropathy Screening Instrument) [19], if available over a relevant time period, could be assessed. Similar cohorts covering a period of time with so many changes in medical management are, however, uncommon.

In conclusion, we have shown significant period effects in the prevalence of PDR, gross proteinuria and peripheral neuropathy in a cohort of persons with T1D, explained largely but not completely by changes in HBA_1c_, BP, and other common risk factors for the complications of T1D that we were able to examine in this cohort. Other changes in medical management and lifestyles factors, including lipid management and patient and physician education about the importance of timely treatment and regular review, might be contributing. Technological advances in methods of achieving glycemic control, and newer forms of insulin and BP medication, are likely to show their greatest impact through the changes in HbA1c and BP levels measured and included in our models. They might, however, be having an effect beyond these measurements. The role of these and additional confounders warrants further exploration in order to gain a better understanding of which interventions have been most effective for reducing the prevalence of these complications in T1D.
